# Complete Genome Sequences of Two Novel *Puma concolor* Foamy Viruses from California

**DOI:** 10.1128/genomeA.00201-12

**Published:** 2013-03-21

**Authors:** Timo Kehl, Anne Bleiholder, Florian Roßmann, Sebastian Rupp, Janet Lei, Justin Lee, Walter Boyce, Winston Vickers, Kevin Crooks, Sue VandeWoude, Martin Löchelt

**Affiliations:** a German Cancer Research Center, Research Program Infection and Cancer, Heidelberg, Germany; b Colorado State University, Fort Collins, Colorado, USA; c University of California, Davis, California, USA

## Abstract

We report two complete foamy retrovirus (FV) genomes isolated from *Puma concolor*, a large cat native to the Americas. Due to high overall genetic relatedness to known feline foamy viruses (FFVs), we propose the name *Puma concolor* FFV (FFV_Pc_). The data confirm that felines are infected with distinct but closely related FVs.

## GENOME ANNOUNCEMENT

Foamy viruses (FVs), also known as syncytial or spuma(retro) viruses, represent the subfamily *Spumaretrovirinae* within the *Retroviridae* and are characterized by a complex genetic organization, distinct molecular biology, ability to cross species barriers, and strong ancient coevolution with their authentic hosts (1–4). Although FVs are often highly prevalent in their respective host populations, there is no obvious disease associated with FV infections, even in zoonotically infected humans (5, 6).

Here, we report the complete consensus sequences of two FV genomes, isolated from free-ranging American pumas (*Puma concolor*), displaying high genetic relatedness to other known feline FV (FFV) isolates, thus designated FFV_Pc_ (accession numbers KC292054 and KC292055). FFV_Pc_ genomic sequences were amplified by nested PCR from peripheral blood leukocyte DNA of animals displaying FFV Gag seroreactivity in enzyme-linked immunosorbent assay (ELISA) and immunoblotting (to be described elsewhere). The animals, designated X102 and X103, were sampled on the same day in San Diego County, California, and are juvenile (<1 year old) male sibling cubs, still in contact with their dam at the time of capture. Initial primers for *gag*, *env*, and *bel* were derived from the FFV serotype FUV (7), while subsequent PCRs were based on the generated FFV_Pc_ sequences. Two to eight individual amplicons were sequenced per animal per genomic region using Sanger sequencing with the ABI 3730xl system. Sequence alignments and consensus sequences were generated by a progressive pairwise global alignment method using Geneious 6.0 (8). The overall genetic organization in both the structural (*gag*, *pol*, and *env*) and accessory (*tas/bel1* and *bel2/bet*) genes and the apparent conservation of canonical FV splice sites in both isolates are consistent with the common characteristics of FVs (9).

Only six nucleotides in *pol* and *env* differed between the two FFV_Pc_ isolates. The three polymorphisms in *pol* are silent, while variations in *env* cause the amino acid changes N265D, I531V, and F536L. In all six nucleotide polymorphisms, FFV_Pc-X102_ is identical to FFV FUV. Comparisons to domestic cat FFV sequences revealed overall sequence similarity of 94.4 to 94.5% (7, 10–13).

Importantly, both FFV_Pc_ isolates are highly related to FFV FUV, which has never before been observed in nondomestic felids (11). Clear genetic differences to known FVs from other felines and comparably high seroprevalence in pumas living in the wild argue that the novel FFV_Pc_ isolates are not the end product of current interspecies transmission events but rather an indigenous puma FV. In contrast to *in vitro* studies of primate, feline, and bovine FVs ([9]; T. Hechler and M. Löchelt, unpublished data), we did not find evidence of FFV_Pc_ cDNAs lacking the FV *bet* intron or other splice events in the *env*-*bel* region from DNA directly derived from infected pumas.

In summary, the two FFV_Pc_ genomes presented here confirm the concept that felines, like primates, harbor highly related but distinct FVs. Sequence variation between both isolates is very low, as both viruses were probably derived from the cubs’ dam, from whom blood samples were unavailable.

### Nucleotide sequence accession numbers. 

The genomic FFV_Pc_ sequences have been deposited in the GenBank database under accession no. KC292054 for FFV_Pc-X102_ and accession no. KC292055 for FFV_Pc-X103_.
